# CULTURALLY RESPONSIVE INTERVENTIONS FOR OLDER ADULTS: FROM DEVELOPMENT TO ADAPTATION

**DOI:** 10.1093/geroni/igad104.1020

**Published:** 2023-12-21

**Authors:** Manka Nkimbeng, Quinton Cotton, Lauren Parker

## Abstract

Culture is an important aspect of health and healthcare because it influences perceptions and health seeking behaviors, but many health interventions are developed without consideration of the cultural needs or do not include the voices of the of the target population. Yet culturally responsive interventions have been proven to be more effective than generic interventions. Opportunely, there is a growing understanding of the need for culturally responsive interventions but limited understanding of the process. This symposium will offer an overview of the process of developing culturally responsive interventions. It will present three unique projects that are in different stages and on different levels on the continuum of culturally responsive interventions and programs. African MADE is a culturally tailored dementia education program that was developed with the African immigrant community, while “Community-Organization Based Intervention Planning for Older Latine Adults: Lessons Learned” describes the co-development process employed in the design of an intergenerational respite program for older adults within the Latine community. Finally, we will present findings of a feasibility study of an evidence based intervention that was adapted for African American women with pain and depression.

